# Mechanical and Durability Properties of Rubberized Sulfur Concrete Using Waste Tire Crumb Rubber

**DOI:** 10.3390/ma17215269

**Published:** 2024-10-30

**Authors:** Okpin Na, Giyeol Lee

**Affiliations:** 1 Hyundai E&C, Technology Research Center, Seoul 03058, Republic of Korea; nao@hdec.co.kr; 2 Department of Landscape Architecture, College of Agriculture and Life Science, Chonnam National University, Gwangju 61186, Republic of Korea

**Keywords:** rubberized sulfur concrete, dry and wet mixing process, toughness index, durability

## Abstract

The use of rubber crumbs provides a viable solution for alleviating the disposal problem of waste tires. In this study, rubberized sulfur concrete (RSC) was researched to investigate the optimal mixture proportion and to improve the mixing process in terms of compressive strength and durability performance. For the mixture of the RSC, sand, rubber particles, and micro-filler were adopted as aggregates and sulfur was used for the binding material. Moreover, two mixing processes were applied: the dry mixing process and the wet mixing process. Based on the test results, the increment of rubber particles in the mixture led to a decrease in the compressive strength for both the dry and wet mixing processes. To minimize the voids between the sand and rubber particles, the micro-filler was used at 5% of the total volume. The amount of sulfur varied slightly depending on the mixing process: 30% sulfur for the dry mixing process and 34% sulfur for the wet mixing process, respectively. Consequently, compared to the dry mixing process, the wet mixing process increased the bonding force between sulfur and rubber powder due to the simultaneous heating and combining. In toughness, the wet mixing process demonstrates a 40% higher energy absorption capability compared to the dry mixing process. For the durability performance of the RSC, the mixture with 20% rubber particles produced using the wet mixing process exhibited better corrosion and freeze–thaw resistance.

## 1. Introduction

The use of rubber crumbs provides a viable solution for alleviating the disposal problem of waste tires. Recently, the number of registered domestic automobiles in Korea has approached one per two people, leading to a high interest in recycling waste tires. Waste tires are reused through three reproducing methods: original form, pyrolysis, and the crumb powder form. In the construction field, the crumb powder manufacturing method, particularly using cryogenic grinding, is prevalent, producing a 200 μm fine powder. The composition of these waste tires, excluding a minuscule amount of moisture, consists of volatile matter and fixed carbon. Moreover, the primary raw material in tire manufacturing is styrene–butadiene rubber (SBR). The sulfur material, a source of harmful gas, comprises about 0.97% [1].

The utilization of waste tires has been extensively researched in Korea and abroad since the mid-1990s. For enhancing the performance of concrete pavement in Korea, approximately 7% waste tire powder in the concrete mix was used [2]. Also, to evaluate the deformation resistance of the asphalt concrete pavement, the research of waste tire particles was conducted. The results concluded that the addition of CRM (Crumb Rubber Modifier) and the use of polymer-modified asphalt mixtures have a significant effect on improving the deformation resistance of the materials. Furthermore, although the tensile strength of conventional asphalt concrete was superior, the other properties, such as the ductility, the maximum vertical deformation, and the resilience modulus, were much better [3]. However, despite the overall remarkable material properties, the bonding issue between the crumbed waste tires and the cementitious matrices of the asphalt concrete have come out. To enhance the adhesion between the cement mortar and the crumb rubber powder, styrene–butadiene rubber latex (SBR) and resin powder were used, and their mechanical and durability properties were investigated [4,5,6]. Recently, to reduce the spalling of high-strength concrete under a fire condition, waste tire particles with metakaolin were applied. In the research results, metakaolin improved the fire resistance of the concrete itself and the waste tires had significant effects on the explosion reduction due to the absorption of the internal expansion [7,8]. Furthermore, rubberized concrete has been studied in the utilization of surface-treated rubber particles with silane or organosulfur to improve the mechanical properties [9,10,11,12,13,14,15,16,17,18].

On the other hand, some researchers have studied sulfur concrete. For sulfur to be used as a binder, it needs to be melted down at temperatures ranging from 130 to 146 °C. The manufacturing process of sulfur concrete is similar to that of asphalt concrete. The basic mixture materials of sulfur concrete consist of sulfur, aggregates, and mineral admixture. Sulfur concrete has excellent properties such as high strength, friction resistance, and chemical resistance, along with thermoplastic properties. In addition, it can be pulverized for recycling as well as molded without the degradation of its mechanical properties [19,20,21,22,23,24]. Currently, sulfur concrete is used in chemical plants and port facilities that require durability and chemical resistance. Another application of sulfur concrete could be in extreme construction environments such as the polar region and the lunar surface, due to its non-exothermic reaction [25,26].

Recently, to improve the fire resistance and ductility of sulfur concrete, glass fiber was adopted [27]. However, even though many researchers have studied sulfur concrete, the effect of incorporating waste rubber particles has not been sufficiently investigated.

The purpose of this study is to investigate the optimal mixture proportion and to improve the mixing process in terms of compressive strength and durability performance. To optimize the mixture proportion of the RSC, sand, rubber particles, and micro-filler as aggregates were adopted, and sulfur was used for the binding material. For the optimal mix design, initially, the effects of the sulfur amount, the rubber content, and the micro-filler were investigated in accordance with the dry mixing processes. Next, the optimal mix design was adjusted with the wet mix process, and then, the toughness and durability performances were evaluated.

## 2. Experimental Detail

### 2.1. Materials

**Figure 1:**
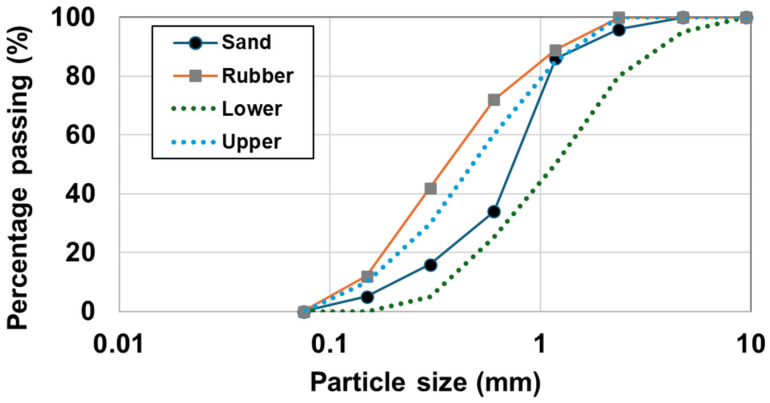
Sieve analysis of sand and rubber particles.

Concrete can consist of three physical phases including aggregate, paste, and porosity. Sulfur, sand, rubber particles and micro-fillers such as fly ash were used as constituents for the sulfur concrete. Therefore, we refer to the concrete as rubberized sulfur concrete. It is important to note that sulfur served as the binder in the mixing process, and no hydration reaction occurred because no cement or water was used in the mixture. The details of the dry process and the wet process will be introduced later. The specific gravity of river sand as a fine aggregate was 2.6 g/cm^3^ with a fineness modulus of 2.63 and water absorption of 2.5%, respectively. The rubber particle was produced by JaiTire Industries Inc. (Denver, CO, USA). The particle size of the rubber is illustrated in Figure 1. The bulk density and specific density of the rubber particles were 0.5 g/cm^3^ and 1.0 g/cm^3^, respectively. The sulfur was from Test Mark Industries, Pennsylvania. Figure 1 shows the size distributions of the sand and rubber particles, and Table 1 indicates the physical and mechanical properties of the sulfur, which was used for the binder in this study.

### 2.2. Mix Design

To determine the optimized mixing proportion for rubberized sulfur concrete (RSC), the mixture proportion was indicated according to the volume ratio of the materials as shown in Table 2. Firstly, the initial volume proportion of the sulfur, 16%, was determined based on the apparent workability of N1 and N2. Then, to obtain further workability, the sulfur content was increased to 26%, as shown in N3 to N8, and the sand was replaced by rubber particles up to 50% of the volume ratio of the sand. The micro-filler in the asphalt mix plays an important role in the stiffening and toughening of a binder [35,36].

**Figure 2:**
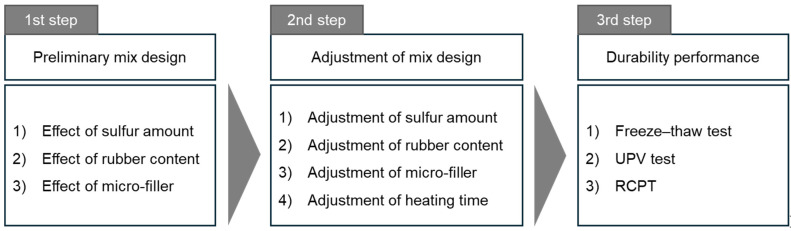
Decision-making process for optimal mix design.

The micro-filler was also used to improve the mechanical properties in the mix designs N9 to N16. The initial volume of the micro-filler was set at 10% of all the compounds, except the sulfur. In N9 to N14, the amount of rubber powder in the place of sand increased up to 50% by volume. In addition, in N15 and N16, an amount of sulfur was added to investigate the effect of the binder and the micro-filler. In Figure 2, the process for selecting the optimal mix design conditions in this study are illustrated in three stages, with detailed variables considered at each step.

### 2.3. Mixing Process

**Figure 3:**
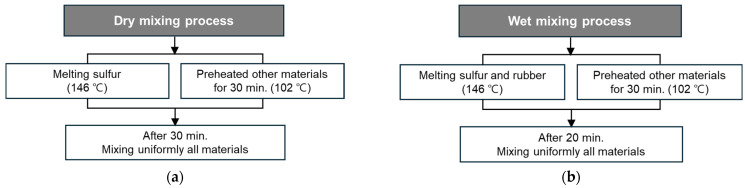
Two types of mixing processes. (**a**) Dry mixing process. (**b**) Wet mixing process.

The mixing process of the RSC can be divided into a dry mixing process and a wet mixing process as shown in Figure 3. For the dry process, the sulfur was placed into a pot and heated first. The suppliers recommended a melt temperature range for the sulfur from 135 °C to 146 °C. The heating temperature of the mixing process was suitable at 146 °C and the heating time for the dry process was about 30 min until the sulfur had completely melted to a slurry. The other materials (the rubber particles, the sand, and the Portland cement) were mixed uniformly with a hand paddle. They were then preheated to about 95 °C in an oven for 30 min and then were added to the hot pot. For the wet process, the sulfur and rubber particles were put together into the pot and heated. The heating time varied to find the specific time that produced the highest strength in this study. The other materials (other than the sulfur and the rubber particles) were preheated to about 102 °C in an oven for 30 min before being added to the pot.

One can see that the main difference between the wet and the dry process is that only the sulfur is pre-heated in the dry process, while the sulfur and the rubber particles are pre-heated together in the wet process. Two similar processes are commonly used in the asphalt industry, where the asphalt can be heated first (dry process) or heated together with aggregates (wet process). It was observed that the two processes have significantly different effects on various properties of the asphalt concrete. The two processes were used in the present study to examine if they have any significant effect on the properties of the RSC.

After adding all the materials to the pot, the actual temperature of the mixture was lower than the original melting temperature. The real temperature of the heated mixture in the pot was hard to measure because the mixture was in a semi-fluid and semi-solid state. The mixture was continually heated in the pot until the mixture became visually uniform and semi-fluid.

### 2.4. Specimen Preparation and Test Methods

Making sulfur concrete is not a common practice in the concrete industry. Therefore, the specimen preparation procedures used in this study are described in detail. Referring to ASTM C 1312-97 (Making and Conditioning Chemical-Resistant Sulfur Polymer Cement Concrete Test Specimens in the Laboratory), all the materials were weighed accurately [37]. Then, two different procedures, a dry process and a wet process, were used to prepare the rubberized sulfur concrete. The molds used for making the RSC samples were 75 mm diameter and 150 mm height cylinders. If the size of a specimen is bigger than 75 mm by 150 mm, the preparation of the specimen is not easy because the sulfur is hardened very fast. One batch contained six specimens for each design mix. The inside surfaces of the molds were coated slightly with mineral oil. For measuring the axial deformation, the gauge length of the axial extensometer was 100 mm. Before testing for the compressive strength, the RSC specimens were capped like regular Portland cement concrete cylinders. Compressive strength tests were performed using a universal testing machines after seven days.

## 3. Results and Discussion

### 3.1. Effect of Sulfur Amount with Rubber Particles and Micro-Filler

There have not been sufficient research results for the RSC used for sulfur as a binder and rubber particles obtained from used tires as an aggregate. Therefore, the proper range of sulfur for the RSC has not been revealed in the literature. Thus, in this study, the initial sulfur amount of 16% was adopted for the mixture proportion of the RSC and the sulfur amount was increased to 30%, according to the visual inspection of the workability and mixing appearance.

**Figure 4:**
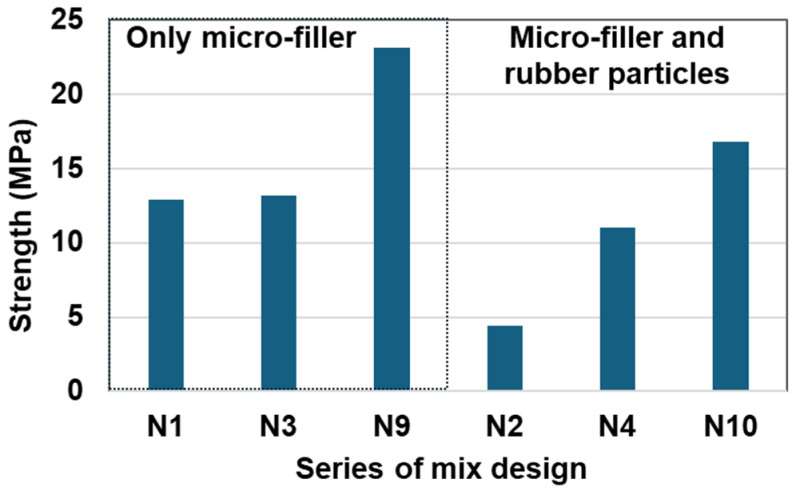
Effect of sulfur amount on rubber particles and micro-filler on compressive strength.

Table 3 and Figure 4 show the effect of the sulfur amount on the compressive strength of the RSC, in terms of rubber particles and micro-filler. N1, N3, and N9 contained no rubber and had three different sulfur contents, 16%, 26%, and 30%, respectively (see Table 3). N2, N4, and N10 contained the same amount of rubber particles, a 10% volumetric ratio of rubber to the aggregate (aggregate includes rubber, micro-filler, and sand), as well as had three different sulfur contents. 

From N1 and N2, one can see that with the same amount of sulfur binder, when a part of the natural aggregates was replaced by rubber particles, the strength reduced by about a third. In fact, when the specimens of N1 and N2 were mixed, it was observed that the rubber aggregates were only partially covered by the sulfur cement and many voids existed in the matrix, which caused difficulty in mixing and placement. Therefore, sulfur content is a very important mix design parameter for the RSC. Without a sufficient sulfur amount, the strength of the RSC will be reduced by the soft rubber particles (as shown in Figure 4) as well as by the low-quality sulfur paste matrix. N9 illustrates the better mixture with a sufficient sulfur amount and micro-filler.

In order to study the effect of sulfur cement content on the compressive strength of the RSC, the volumetric sulfur amount of N3 and N4 was increased to 63%, compared to the sulfur amount of N1 and N2. The volumetric ratio of rubber to sand for N1 and N3 was the same (0:100) and so was the ratio of N2 and N4 (10:90), as shown in Table 3. From Figure 4, one can see that with the same amount of rubber replacement (about 7%), the strength reduction from N3 to N4 with a 26% sulfur amount is not as high as that from N1 to N2 with 16% sulfur. This means that there must be a sufficient amount of sulfur as the binder, and 16% is not sufficient. Based on the present test data, the sulfur content of N3 and N4 may be considered sufficient, which is 26%. With sufficient binder, the addition of rubber particles still reduces the strength of the RSC, which is the strength reduction observed in N3 and N4. As shown in Table 3 and Figure 4, in case the rubber particles and micro-filler are mixed additionally as aggregates, the sulfur binder shall be increased for workability.

**Figure 5:**
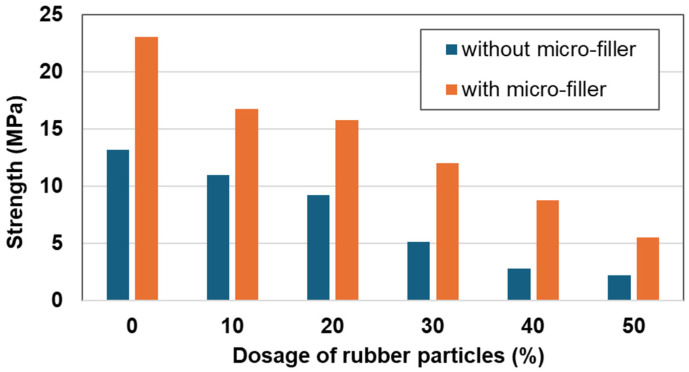
Effect of rubber particles with and without micro-filler on compressive strength.

Based on the results of Figure 4, the effect of the rubber particles is investigated in Table 4. The test series of N3 to N8 is demonstrated for the increment of rubber particles and that of N9 to N14 is added with a micro-filler of 7%. In Figure 5, it is very clear that, with the increase in rubber content, the compressive strength of the RSC decreases considerably. This result is expected because the strength of the rubber particles is much lower than that of the sulfur concrete (without rubber). One important result shown in Figure 5 is that with the addition of micro-filler, the compressive strength of the RSC is increased dramatically. In particular, for the high rubber contents from 30 to 50%, the compressive strengths of the RSC with micro-filler are doubled compared to those without the micro-filler. This means that the micro-filler reduces the porosity of the RSC and improves the strength of the material significantly.

The two test series in Figure 5 are almost parallel with almost the same negative slope, which means that the micro-filler can improve only the properties of the sulfur matrix and cannot compensate the strength loss due to the addition of the rubber particles. It also means that the improvement of the matrix by the micro-filler is not affected by the rubber particles. In other words, the strengthening of the matrix by micro-filler and the reduction in strength by the added rubber particles are two independent mechanisms.

**Figure 6:**
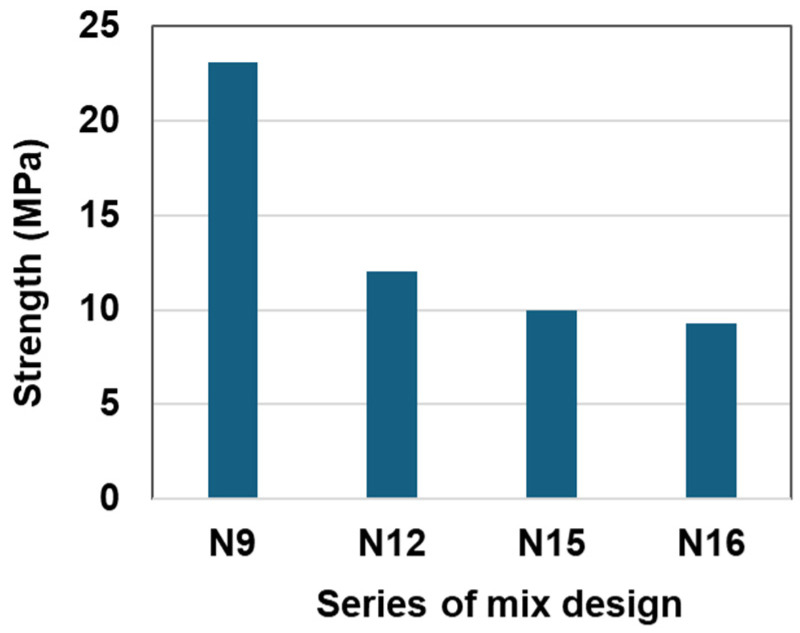
Effect of sulfur amount with and without rubber particles on compressive strength.

Based on the results of N9 in Figure 4 and Figure 5, additional tests were prepared to investigate the effect of sulfur content with micro-filler and rubber particles. Figure 6 indicates the strength comparison among specimens N12, N15 and N16 in Table 5. For these test series, the volumetric ratio of rubber to aggregate (aggregate includes cement, rubber, and sand) was kept as a constant 30%, while the sulfur contents were different, from 30% to 37%. The sulfur contents of N12, N15, and Nl6 were 1:1.1:1.23, respectively. The strength ratios of the three specimens were 1:0.84:0.78. It is important to see that the advantages of increasing the sulfur content are the compressive strength and workability of the RSC only in a certain range. If the sulfur content is beyond a critical value, the strength of the RSC will decrease. This indicates that the sulfur amount should be controlled within a proper range because excessive sulfur in the mixture has an adverse effect on its strength. Based on the results obtained in this study, the starting value for the sulfur proportion is about 30% of the total volume. A sulfur content that is less than the critical value may result in low workability, difficulty in mixing and placement, and low strength; a sulfur content that is higher than the critical value may lead to a reduction in the strength of the RSC.

### 3.2. Effect of Micro-Filler

**Figure 7:**
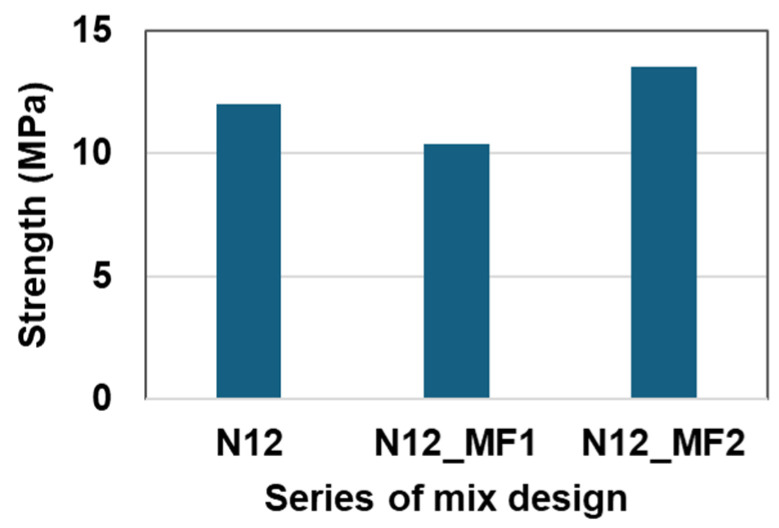
Effect of micro-filler on compressive strength.

Table 6 was designated for the effect of micro-filler on the compressive strength of RSC. The content of the micro-filler was fixed at 7% of the total volume. In order to find the optimal micro-filler content, several micro-filler contents of 3% and 5% were applied to additional RSC mixture series and tested for their compressive strength. The mixture proportions of N12, N12_MF1, and N12_MF2 with different micro-filler contents are listed in Table 6 and the test results are shown in Figure 7. It can be seen that N12_MF2 is 13% higher than N12 in compression strength. The micro-filler content of N12_MF2 is about 5% of the total volume. Therefore, 5% micro-filler by volume can be considered the best content in this study.

**Figure 8:**
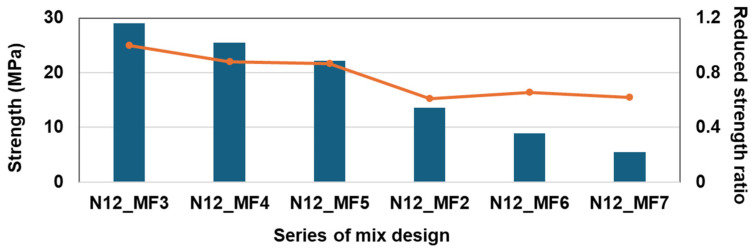
Effect of sulfur amount on rubber particles and micro-filler on compressive strength and reduced strength ratio.

While fixing the micro-filler content to 5%, the effects of the rubber content on RSC strength with increments of rubber content were investigated. The mixture series from N12_MF2 to N12_MF7 had the same sulfur and micro-filler contents and different rubber contents ranging from 0% to 50% of the rubber to aggregate ratio. Figure 8 shows the effect of the rubber dosage on the compressive strength of the RSC. It can be seen that the strength decreases when the rubber content increases, similar to the trend shown in Figure 5. The difference between Figure 5 and Figure 8 is the micro-filler content. In Figure 8, among the mixture proportions of N12_MF2 to N12_MF7, the lowest reduced strength ratio is N12_MF5, with 20% rubber particle and 5% micro-filler.

### 3.3. Effect of Wet Mixing Process

As previously mentioned regarding RSC, the main factor influencing compressive strength is the micro-filler, which fills the voids and gaps between the aggregates such as the sand and rubber particles. Another significant factor affecting mechanical properties is the mixing process, which can be either dry or wet. The differences between these two mixing processes were described in the previous chapter. To explore the differences between the dry and wet mixing processes, the baseline mixtures of N12 and N12_MF2 were used for comparison. For the wet mixing process, a series of mixtures from W1 to W5 was proposed, with the total aggregate content fixed at 70% and the sulfur content increased, as shown in Table 7.

**Figure 9:**
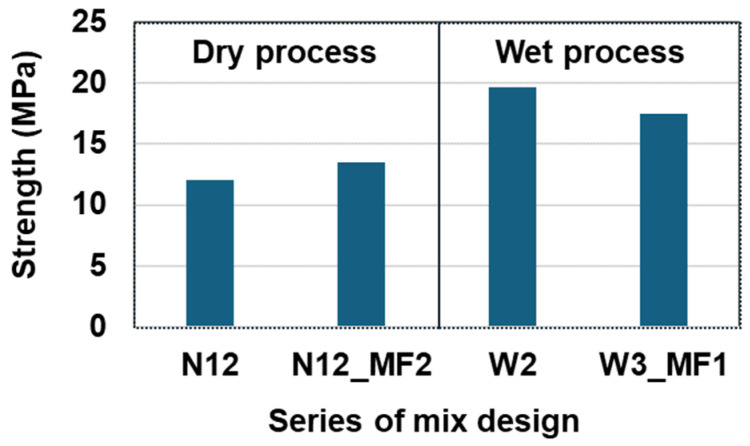
Difference in compressive strength depending on the dry and wet mixing process.

First of all, Figure 9 illustrates the test results comparing the dry mixing process of N12 and N12_MF2 with the wet mixing process of W2 and W3_MF1. Based on these results, the wet mixing process demonstrates better performance than the dry mixing process when using the same mixture proportions. That is, the compressive strength of N12 is 64% higher than that of W2 because the melted sulfur and rubber particles were mixed together more effectively, resulting in better bonding with fewer voids and gaps between the sulfur matrix and the rubber particles.

**Figure 10:**
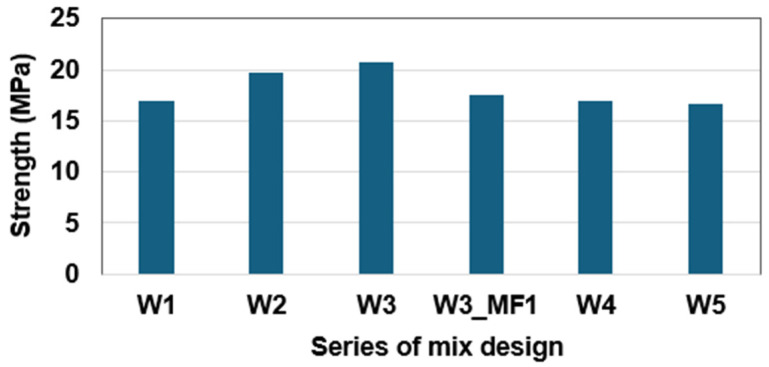
Effect of compressive strength regarding the dosage of rubber particles.

In Figure 10, it can be seen that as the sulfur content increased from 32% to 37%, the compressive strength initially increased and then decreased. The maximum compressive strength, just above 20 MPa, can be observed in W3 in Figure 10. This mixture has a sulfur content of approximately 34% by total volume, which can be considered the optimal sulfur content and will be used in the following sections.

### 3.4. Modified Mixing Process

**Figure 11:**
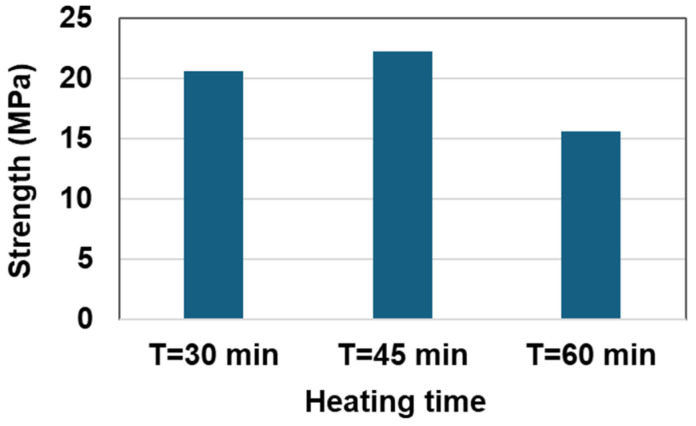
Effect of compressive strength depending on the heating time.

**Figure 12:**
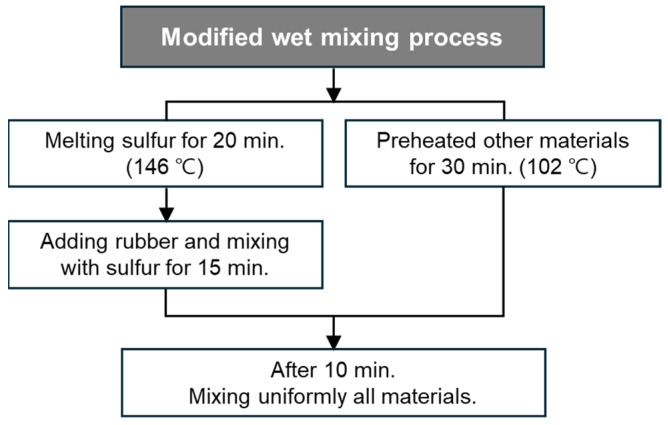
Proposed modified wet mixing process.

In the wet mixing process, heating time is a crucial factor, since sulfur is melted together with rubber. The optimal temperature for melting sulfur powder ranges from 135 °C to 146 °C; in this study, 146 °C was used. The heating durations were set to 30, 45, and 60 min. The compressive strength was measured for each heating duration at 146 °C with the mix conditions of W3. Figure 11 illustrates how strength varies with heating duration, showing that the highest strength was achieved with a heating duration of 45 min. Prolonged heating beyond this duration led to sulfur burning, which resulted in a decrease in strength. Consequently, a modified wet mixing process with the optimal heating duration was recommended, and the detailed procedure is illustrated in Figure 12.

**Figure 13:**
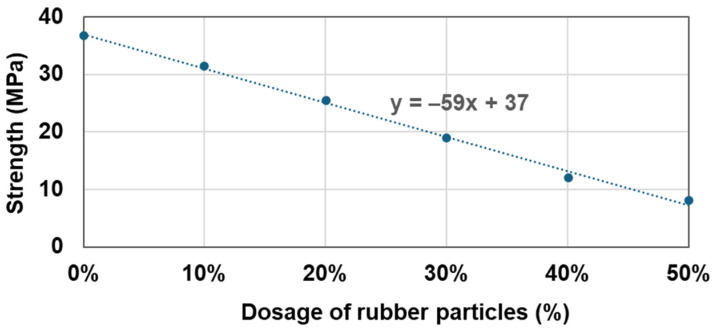
Regression analysis of mix design for RSC regarding the dosage of rubber particles.

Table 8 presents the results of compressive strength measurements with varying amounts of rubber particles from 0% to 50% under the optimal wet mixing conditions. The data indicate that the strength decreased almost linearly with the increasing rubber powder content. The relationship between the strength and the rubber powder content can be predicted using the formula shown in Figure 13.

### 3.5. Toughness of RSC According to Two Mixing Process

**Figure 14:**
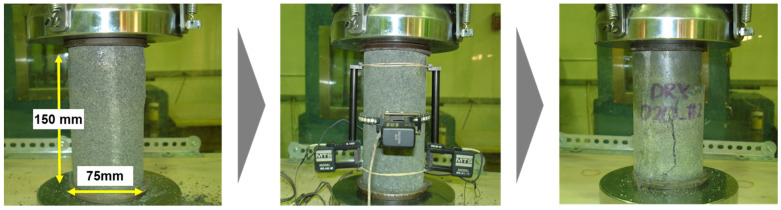
Set-up of RSC specimen with strain gauges.

**Figure 15:**
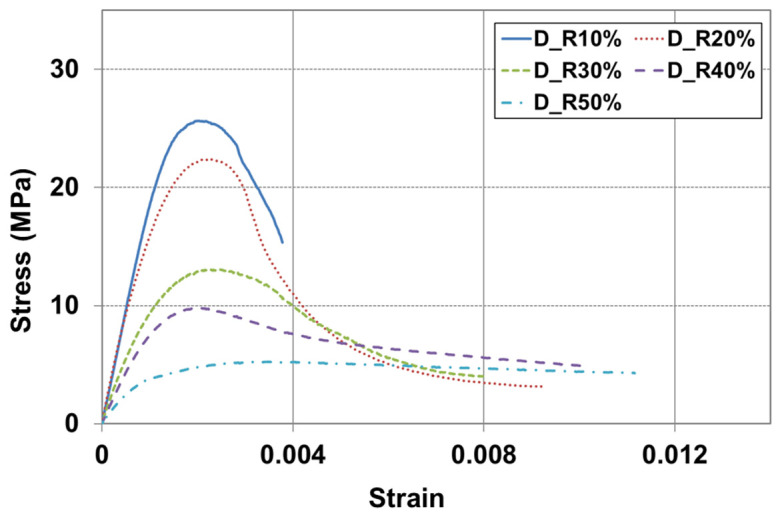
Stress–strain curve on dry mixing process.

**Figure 16:**
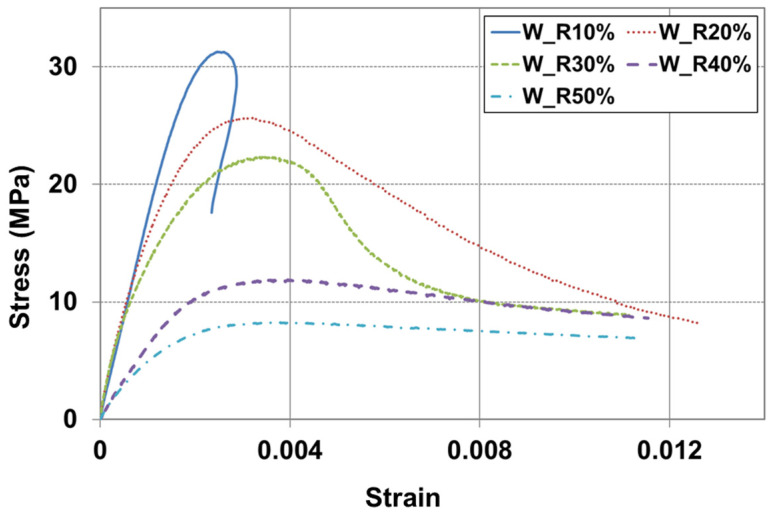
Stress–strain curve on wet mixing process.

The toughness of a material can be determined from the area under the stress–strain curve. This allows for an understanding of the material’s brittleness and ductility, as well as its resistance to fatigue fracture. Figure 14 illustrates the setting up for a compressive strength test, using MTS axial extensometers to obtain the stress–strain curve. Each extensometer is 114 mm long and it is attached to both sides of the specimen. Figure 15 and Figure 16 show the stress–strain curves for RSC specimens with varying rubber powder content (10% to 50%), tested under the optimal mix condition, using both dry and modified wet mixing processes. Under the same mixture proportion, the RSC specimens produced by the wet mixing process generally exhibited higher stress and longer strain.

**Figure 17:**
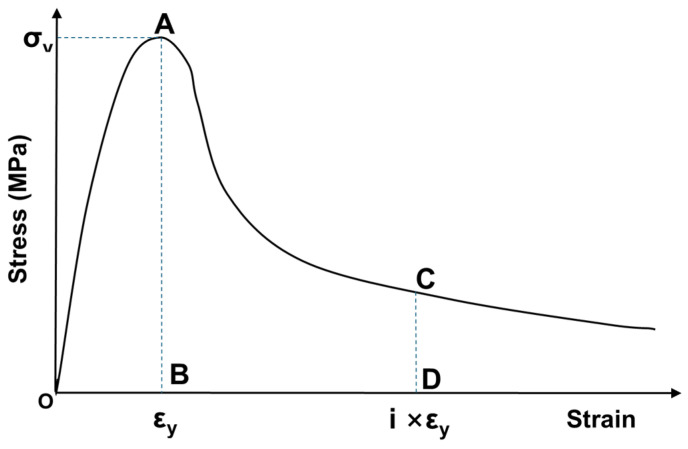
Toughness index from stress (σ)–strain (ε) curve.

**Figure 18:**
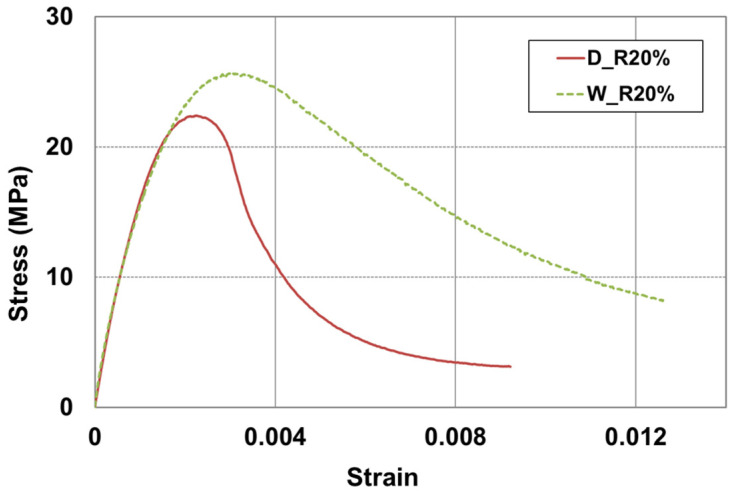
Comparison of toughness between D_R20% and W_R20%.

Figure 17 illustrates the method for calculating the toughness index. The toughness index can be expressed as the ratio of the area OAB up to the initial crack to the area OACD up to the comparative deformation [38]. Figure 18 shows only the comparative result where 20% rubber particle was mixed, for the purpose of determining the toughness index of the RSC according to their manufacturing process, as seen in Figure 15 and Figure 16. In Figure 18, the toughness index is represented as the area ratios for strains of 0.8% and 1.2%, which are four times the yield strain. For the calculation of the area underneath the strain-stress curve, Image J (ver. 1.46), a public domain Java-based image processing and analysis program, was used [39]. As shown in Table 9, it can be observed that the method using the wet mixing process exhibited a higher energy absorption capability of 40% and better resistance to fatigue impact compared to the dry mixing process.

### 3.6. Durability Performance of RSC

#### 3.6.1. Freezing–Thawing Resistance Test

**Figure 19:**
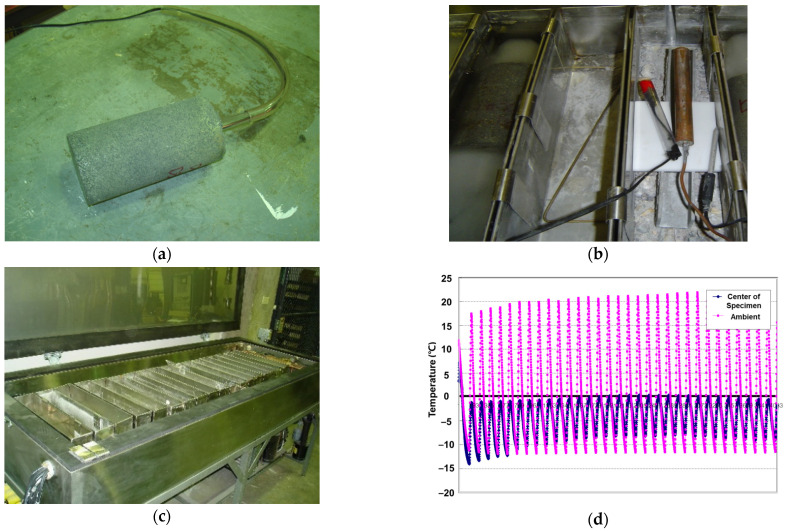
Temperature sensors and test equipment. (**a**) Set-up the sensor in the center of the specimen (**b**) Sensor for measuring the ambient temperature inside the chamber (**c**) Freezing–thawing chamber (**d**) Freezing-thawing temperature cycles.

The purpose of this test is to evaluate the resistance of freezing and thawing in water, as outlined by ASTM C666 [40]. The freezing-and-thawing chamber is equipped with refrigeration and heating systems, along with controls, to continuously and automatically generate reproducible cycles that meet the specified temperature requirements. In the chamber, cylindrical RSC specimens with 75 mm diameter by 100 mm height were used and they were cast with a modified wet mixing process. For monitoring the temperatures inside the chamber and in the center of the specimen, temperature sensors were installed as shown in Figure 19a,b. The temperature range in the center of the specimen was from 0 °C to −13 °C and the ambient temperature range was about 20 °C to −12 °C as shown in Figure 19d.

**Figure 20:**
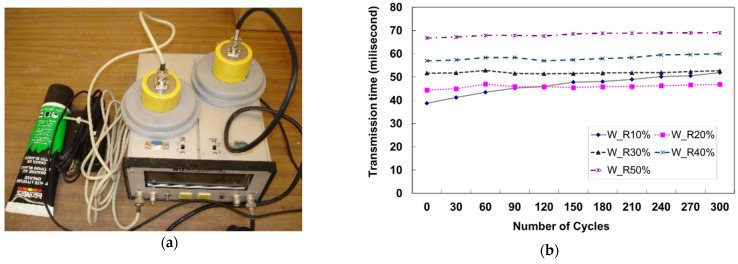
UPV test after F-T cycles. (**a**) Ultra-sonic pulse velocity tester (**b**) traveling time with UPV.

This freezing–thawing test (F-T test) was conducted for 300 cycles and one complete cycle took approximately 3 h. After 30 cycles, the transmission time was measured with an ultrasonic pulse velocity (UPV) tester as shown in Figure 20a. According to the UPV results shown in Figure 20b, the transmission time through the RSC specimen increased slightly. Specifically, for the case with 10% rubber particle replacement of the total aggregate, the variation in transmission time was significantly higher compared to the other cases. The accumulation of the freezing–thawing cycles resulted in damage to the specimen, particularly with lower rubber dosages. In other words, rubber particles are a primary factor contributing to increased micro-damage in RSC.

**Figure 21:**
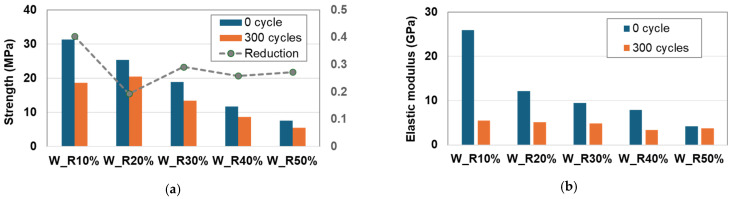
Reduction in mechanical properties. (**a**) Compression strength after F-T cycles. (**b**) Elasticity after F-T cycles.

To investigate the effect of the mechanical properties under freezing–thawing conditions, a compression test was conducted before and after 300 freezing–thawing cycles. As shown in Figure 21a, the maximum reduction in compressive strength was 40% for the W_R10% mixture. Conversely, the strength reduction for the W_R20% mixture was lower compared to the other mixtures, indicating that rubber particles were effective in mitigating the contraction and expansion of the RSC under freezing and thawing conditions. Furthermore, Figure 21b shows that the elastic modulus of the RSC decreased significantly, by up to about 80%. This suggests that rubber particles may enhance the durability performance of RSC.

#### 3.6.2. Rapid Chloride Penetration Test

**Figure 22:**
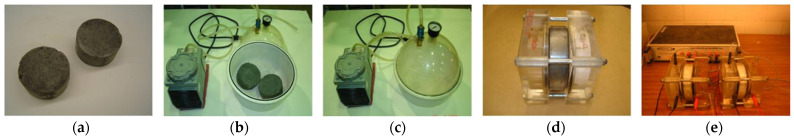
Procedure of RCPT. (**a**) Preparation of specimens. (**b**) Set-up with pump. (**c**) Vacuuming. (**d**) Assembling test cell. (**e**) Proove-It tester.

A rapid chloride penetration test was conducted to evaluate the resistance of rubberized sulfur concrete to chloride ion penetration in moist and chemically aggressive environments, in accordance with ASTM C1202 [41]. The RCP test was conducted using cylindrical specimens with a diameter of 100 mm and a height of 50 mm, which were cured for 7 days after casting. The experimental apparatus known as the PROOVE-It tester was used, as illustrated in Figure 22e.

Before conducting the RCP test, the cylindrical specimens were prepared by sealing the cylinder walls with silicone and allowing it to dry for 1 h. This coating was intended to prevent fluid leakage from the sidewalls and to ensure a linear flow of electrical current through the specimen. The specimens were then subjected to a vacuum for three hours and saturated for 18 h according to ASTM standards. Following this, each specimen was immediately loaded into the Proove-It device. Once loaded, solutions were added to the two test cell containers: one containing a 3% NaCl solution and the other containing 0.3 N NaOH. The testing device then applied a 60-volt potential across the faces of the specimen for six hours. All the procedures of the RCP test are demonstrated in Figure 22a–e. During this period, the apparatus measured the impedance in coulombs throughout the entire duration of the test.

**Figure 23:**
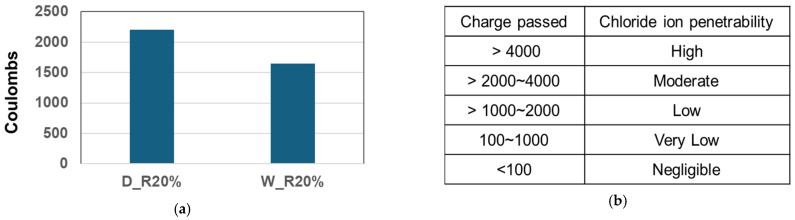
Result of RCPT. (**a**) Comparison of permeability between D_R20% and W_R20%. (**b**) Ion penetrability index as per charge passed.

This test method measures the electrical conductivity of the specimen, which indicates the permeability of sulfur-binding materials. Permeability is determined by the total porosity of the RSC and the connectivity of its pores. For optimal resistance to moisture and aggressive chemicals, the permeability should be minimized. In Figure 23a, the permeability of D_R20% and W_R20% was compared. As noted in the previous chapter, W_R20% was produced using a wet mixing process with 20% replacement of the total aggregate. Based on the test results, the ion penetrabilities of D_R20% and W_R20% were 2200 and 1650 coulombs, respectively. According to Figure 23b, the level of D_R20% was moderate and that of W_R20% was low. This is because the internal organization of W_R20% is likely to be more sophisticated due to the different mixing process.

## 4. Conclusions

This study aimed to determine the optimal mixture proportion for rubberized sulfur concrete (RSC) in terms of compressive strength. For the optimal mix design, initially, the effects of the sulfur amount, the rubber content, and the micro-filler were investigated in accordance with the dry mixing processes. Next, the optimal mix design was adjusted with the wet mix process. Then, the toughness and durability performances were evaluated. For optimizing the mixture proportion of the RSC, sand, rubber particles, and micro-filler were adopted as aggregates and sulfur was used for the binding material. Moreover, two mixing processes were applied: the dry mixing process and the wet mixing process.

Based on the test results, the increment of rubber particles in the mixture led to a decrease in the compressive strength for both the dry and wet mixing processes. To minimize the voids between the sand and rubber particles, the micro-filler was used at 5% of the total volume. The amount of sulfur varied slightly depending on the mixing process: 30% sulfur for the dry mixing process and 34% sulfur for the wet mixing process.

In the wet mixing process, the sulfur was not completely mixed with the rubber particles during the heating time, as the rubber particles tended to be burned before the sulfur melted. Thus, a modified wet mixing process was proposed. Consequently, compared to the dry mixing process, the wet mixing process increased the bonding force between the sulfur and the rubber powder due to their simultaneous heating and combining.

For the toughness of the RSC, the stress–strain profiles of the dry and wet mixing processes were compared using a mixture with 20% rubber particles. The toughness index was represented by the area ratios for strains of 0.8% and 1.2%, which were four times the yield strain. The results show that the wet mixing process demonstrates a 40% higher energy absorption capability compared to the dry mixing process. Therefore, it is determined that RSC can be applied to construction sites where low-strength high-toughness materials are required, such as in road paving or lightweight walls.

For the durability performance of the RSC, a freezing–thawing (F-T) test and a rapid chloride ion penetration (RCP) test were conducted. According to the results of the transmission time, the increment of the F-T cycles led to damage in the RSC specimens, but the rubber particles were able to enhance the durability of the RSC by alleviating the micro-damage. The RCP test was assessed to determine the permeability of the sulfur-binding materials. According to the results of the RCPT, the mixture with 20% rubber particles produced using the wet mixing process exhibited better permeability compared to the dry mixing process.

These findings can be applied to precast products such as bricks, sound-absorbing barriers, and lightweight wall panels. Additionally, RSC is suitable for 3D-printing materials due to its quick setting and early strength development.

## Figures and Tables

**Table 1:** Physical and mechanical properties of sulfur binder [28,29,30,31,32,33,34].

Properties	Curing	Temp. (°C)	Value	Test Method
Compressive Strength (MPa)	2 h	25	34.5	ASTM C617
	48 h	25	48.3	ASTM C579
Tensile Strength (MPa)	48 h	25	4.8	ASTM C307
Flexural Strength (MPa)	48 h	25	712.4	ASTM C580
Density (g/cm^3^)	-	-	1.9	ASTM C905
Coefficient of Thermal Expansion (cm/cm/°C)	-	-	3.8 × 10^−5^	ASTM C531
Water Absorption	-	-	0.2%	ASTM C413
Color	Dark Gray			

**Table 2:** Preliminary mixture proportion of RSC (ratio by volume, %).

No.	Sulfur (%)	Total Aggregate (%)			
			Micro-Filler	Rubber	Sand
N1	16	84	0	0	84
N2	16	84	0	8	76
N3	26	74	0	0	74
N4	26	74	0	7	66
N5	26	74	0	15	59
N6	26	74	0	22	52
N7	26	74	0	29	44
N8	26	74	0	37	37
N9	30	70	7	0	63
N10	30	70	7	7	56
N11	30	70	7	14	49
N12	30	70	7	21	42
N13	30	70	7	28	35
N14	30	70	7	35	28
N15	33	67	7	20	40
N16	37	63	6	19	38

**Table 3:** Mixture proportion of the RSC regarding the effect of sulfur amount.

No.	Sulfur (%)	Total Aggregate (%)			Ratio of Total Aggregate (%)		
		Micro-Filler	Rubber	Sand	Micro-Filler	Rubber	Sand
N1	16	0	0	84	0	0	100
N3	26	0	0	74	0	0	100
N9	30	7	0	63	10	0	90
N2	16	0	8	76	0	10	90
N4	26	0	7	67	0	10	90
N10	30	7	7	56	10	10	80

**Table 4:** Mixture proportion of RSC regarding the effect of rubber particles.

No.	Sulfur (%)	Total Aggregate (%)			Ratio of Total Aggregate (%)		
		Micro-Filler	Rubber	Sand	Micro-Filler	Rubber	Sand
N3	26	0	0	74	0	0	100
N4	26	0	7	66	0	10	90
N5	26	0	15	59	0	20	80
N6	26	0	22	52	0	30	70
N7	26	0	29	44	0	40	60
N8	26	0	37	37	0	50	50
N9	30	7	0	63	10	0	90
N10	30	7	7	56	10	10	80
N11	30	7	14	49	10	20	70
N12	30	7	21	42	10	30	60
N13	30	7	28	35	10	40	50
N14	30	7	35	28	10	50	40

**Table 5:** Mixture proportion of the RSC regarding the effect of the increased sulfur amount and the rubber particles.

No.	Sulfur (%)	Total Aggregate (%)			Ratio of Total Aggregate (%)		
		Micro-Filler	Rubber	Sand	Micro-Filler	Rubber	Sand
N9	30	7	0	63	10	0	90
N12	30	7	21	42	10	30	60
N15	33	7	20	40	10	30	60
N16	37	6	19	38	10	30	60

**Table 6:** Mixture proportion of RSC regarding the effect of micro-filler.

No.	Sulfur (%)	Total Aggregate (%)			Ratio of Total Aggregate (%)		
		Micro-Filler	Rubber	Sand	Micro-Filler	Rubber	Sand
N12	30	7	20	40	10	30	60
N12_MF1	30	3	20	46	4	30	66
N12_MF2	30	5	20	44	7	30	63
N12_MF3	30	5	0	65	7	0	93
N12_MF4	30	5	7	58	7	10	83
N12_MF5	30	5	15	51	7	20	73
N12_MF6	30	5	28	37	7	40	53
N12_MF7	30	5	35	30	7	50	43

**Table 7:** Mixture proportion of RSC regarding the effect of the wet mixing process.

No.	Sulfur (%)	Total Aggregate (%)			Ratio of Total Aggregate (%)		
		Micro-Filler	Rubber	Sand	Micro-Filler	Rubber	Sand
N12	30	7	20	40	10	30	60
N12_MF2	30	5	20	42	7	30	63
W1	32	7	21	41	10	30	60
W2	33	7	20	41	10	30	60
W3	34	7	20	40	10	30	60
W3_MF1	34	5	20	42	7	30	63
W4	36	7	19	39	10	30	60
W5	37	7	19	38	10	30	60

**Table 8:** Mixture proportion of RSC regarding the effect of rubber particles via the wet mixing process.

No.	Sulfur (%)	Total Aggregate (%)			Ratio of Total Aggregate (%)		
		Micro-Filler	Rubber	Sand	Micro-Filler	Rubber	Sand
W3_R0	34	7	0	60	10	0	90
W3_R1	34	7	7	53	10	10	80
W3_R2	34	7	13	47	10	20	70
W3_R3	34	7	20	40	10	30	60
W3_R4	34	7	27	33	10	40	50
W3_R5	34	7	33	27	10	50	40

**Table 9:** Toughness indices of different mixing processes.

No.	B		D		OAB	OACD	Toughness Index
	εy	σy	4εy	σ			
D_R20%	0.0022463	22.12	0.008978	3.16	77,021	205,830	2.67
W_R20%	0.0036945	25.52	0.012141	8.63	125,880	470,602	3.74

## Data Availability

The raw data supporting the conclusions of this article will be made available by the authors on request.
